# Supplementary material for: Studies on the Oxidative Damage of the Wobble 5-Methylcarboxymethyl-2-Thiouridine in the tRNA of Eukaryotic Cells with Disturbed Homeostasis of the Antioxidant System
Source: Int J Mol Sci. 2024 Nov 17;25(22):12336. doi: 10.3390/ijms252212336 (PMC11594727; doi:10.3390/ijms252212336)
Supplement: Supplementary file 1 [file ijms-25-12336-s001.zip › ijms-3299813-supplementary.pdf]

SUPPLEMENTARY MATERIALS

TABLES

Table S1. Yeast cell lines used in the studies, media and culture conditions.

Table S2. Human cell lines used in the studies, media and culture conditions.

Table S3. Sequences of sgRNAs targeting the human *SOD1*, *SOD2* and *Cat* genes, PCR primers designed to detect the introduced mutations.

Table S4. Sequences of PCR primers and Northern blot probes used in γ-toxin assay for yeast $tRNA^{Glu}$ analysis, Sc- *Saccharomyces cerevisiae*, 25s- 25S rRNA (positive control).

Table S5. Modification of HEK293 cells. Efficiency of CRISPR/Cas9 gene editing.

Table S6. The list of nucleoside standards used in studies, molecular formula and UV characteristics.

Table S7. LC-MS/MS analysis conditions (A) LC gradient, (B) TOF MS scanning parameters, (C) TOF MS/MS scanning parameters.

FIGURES

Figure S1. T7 Endonuclease assay for selected clones of modified HEK293 with damaged *SOD2* or *Cat* genes selected for genomic DNA sequencing.

Figure S2. The result of sequencing of genomic DNA isolated from HEK293 cells with the damaged *SOD2* or *Cat* genes. Comparison of the sequences obtained from the modified clones (upper strand) with the original gene sequence (lower strand).

Figure S3. The results of the spotting test to investigate the viability of yeast cells exposed to oxidizing agents for 1 or 2 hours.

Figure S4. Determination of the ROS level in yeast cells after incubation with NaClO., the 2,7 dichloro-dihydrofluorescein diacetate assay.

Figure S5. Visualization of γ-toxin (~27 kDa) protein in SDS-PAGE, gel stained with Coomassie Brilliant Blue dye. Line 1. protein mass standard, Line 2. purified protein.

Figure S6. MTT assay for cancer cells exposed to the oxidative reagents $NaAsO_2$ or NaClO. Evaluation of cell viability as a function of reagent concentration.

Figure S7. ROS generation in the cancer cells exposed to H_2O_2, $NaAsO_2$ or NaClO determined by 2,7 dichloro-dihydrofluorescein diacetate assay.

Figure S8. The natural intracellular ROS level existing in cancer cells, evaluated by 2,7-dichloro-dihydrofluorescein-diacetate assay.

Figure S9. Structural formula of the nucleoside standards used in the LC-MS/MS (MRMhr) studies.

Figure S10. The extracted ions chromatogram (XIC) of the mixture of ten nucleoside standards used in the mass studies.

Figure S11. Standard curves prepared for nucleoside standards.

Figure S12. Identification of mcm5S2U desulfuration products in yeast $tRNA^{Glu}$ by LC-MS/MS, yeast exposed to $NaAsO_2$ or NaClO during culture.

Table S1. Yeast cell lines used in the studies, media and culture conditions.

Cell line	Description	Media and culture conditions
INVSc1	*Saccharomyces cerevisiae*	YPD medium. 30 °C, 200 rpm
M3	*Saccharomyces cerevisiae*	YPD medium. 30 °C, 200 rpm
M3*Δsod1*	*Saccharomyces cerevisiae*	YPD medium. 30 °C, 200 rpm

Table S2. Human cell lines used in the studies, media and culture conditions.

Cell line	Description	Media and culture conditions
HeLa	*cervix epitheral adenocarcinoma*	RPMI-1640, 10% FBS, antibiotics. 37 °C, 5% CO_2
K562	*chronic myelogenous leukemia*	RPMI-1640, 10% FBS, antibiotics. 37 °C, 5% CO_2
MOLT-4	*acute T lymphoblastic leukaemia*	RPMI-1640, 10% FBS, antibiotics. 37 °C, 5% CO_2
A375	*malignant melanoma*	RPMI-1640, 10% FBS, antibiotics. 37 °C, 5% CO_2
A549	*lung carcinoma*	RPMI-1640, 10% FBS, antibiotics. 37 °C, 5% CO_2
A431	*squamous carcinoma*	DMEM high glucose, 10% FBS, antibiotics, 37 °C, 5% CO_2
U87 MG	*glioblastoma*	DMEM high glucose, 10% FBS, antibiotics, 37 °C, 5% CO_2
MCF-7	*breast adenocarcinoma*	DMEM high glucose, 10% FBS, antibiotics, 37 °C, 5% CO_2
HEK293	*embryonic kidney*	DMEM high glucose, 10% FBS, antibiotics, 37 °C, 5% CO_2
HEK293*ΔSOD2*	*embryonic kidney del SOD2*	DMEM high glucose, 10% FBS, antibiotics, 37 °C, 5% CO_2
HEK293*ΔCat*	*embryonic kidney del Cat*	DMEM high glucose, 10% FBS, antibiotics, 37 °C, 5% CO_2

Table S3. Sequences of sgRNAs targeting the human *SOD1*, *SOD2* and *Cat* genes, PCR primers designed to detect the introduced mutations.

**sgRNA**	**Sequence [5′→3′]**	**PCR primers for mutation detection**
**Sod1-sgRNA1**	GTGTGCGGCCAATGATGCAA	Sod1-Tg1-Fw 5′-GTTTGCGTCGTAGTCTCCTG-3′<br>Sod1-Tg1-Rv 5′-CAAGCAAGGGACGAGCACA-3′
**Sod1-sgRNA2**	CTAGCGAGTTATGGCGACGA	Sod1-Tg2-Fw 5′-ACTGGCTAGAAAGTGGTCAGC-3′<br>Sod1-Tg2-Rv 5′-CAGCACTTTGGGAGGTCGAG-3′
**Sod1-sgRNA3**	TTGGAGATAATACAGCAGGT	Sod1-Tg3-Fw 5′-TGTTTAGTGGCATCAGCCCT-3′<br>Sod1-Tg3-Rv 5′-CTACTTCTGTTTT CCAAACTCAG-3′
**Sod2-sgRNA1**	GACGTTCAGGTTGTTCACGT	Sod2-Fw 5′-GCTTTCTCGTCTTCAGCACC-3′<br>Sod2-Rv 5′-AGTAAGGCAAGCTCCTTCGC-3′
**Sod2-sgRNA2**	ATGATCTGCGCGTTGATGTG	
**Sod2-sgRNA3**	TCCAGGGCGCCGTAGTCGTA	
**Cat-sgRNA1**	GATGCAGAGACTCAGGACGT	Cat-Tg1-Fw 5′-GGATGGATCCAGGTGCTTCTT-3′<br>Cat-Tg1-Rv 5′-TCTTCGATGCTCTGCAAGGAAA-3′
**Cat-sgRNA2**	GATGGTAACTGGGATCTCGT	Cat-Tg2-Fw 5′-CACCATAATTCCTGTAAACTTAG-3′<br>Cat-Tg2-Rv 5′-GCCAAAACAACCTGAAAACTGG-3′
**Cat-sgRNA3**	GCATGCAGGACAATCAGGGT	Cat-Tg3-Fw 5′-CCTATCCTGACACTCACCGC-3′<br>Cat-Tg3-Rv 5′-CTCCCTGCTTTTAACTACTGGA-3′

Table S4. Sequences of PCR primer and Northern blot probes used in γ-toxin assay for yeast $tRNA^{Glu}$ analyse, Sc- *Saccharomyces cerevisiae*, 25s- 25S rRNA (positive control).

	Name	Sequence [5'-3']
PCR primers	Sc Fw	5'-TCCGATATAGTGTAACGGCTAT-3'
	Sc Rv	5'-CTCCGATACGGGGAGTCG-3'
	25s Fw	5'-GAAATCTGGTACCTTCGGTG-3'
	25s Rv	5'-GATTCTCACCCTCTATGACG-3'
Northern blot probe	NB Sc $tRNA^{Glu}$	5'-ATAGCCGTTACACTATATCGGA-3'
	NB Sc 25s	5'-GATTCTCACCCTCTATGACG-3'

Table S5. Modification of HEK293 cells. Efficiency of CRISPR/Cas9 gene editing.

genes	*SOD1*			*SOD2*			*CAT*		
sgRNA	sg1-RNA	sg2-RNA	sg3-RNA	sg1-RNA	sg2-RNA	sg3-RNA	sg1-RNA	sg2-RNA	sg3-RNA
Selected cells	300	300	300	300	300	300	300	300	300
Clones	36	43	18	35	39	35	93	31	106
Positive clones	0 positive per 97 tested, CRISPR yield 0%			5 positive per109 tested, CRISPR yield 4.6%			19 positive per20 tested, CRISPR yield 95%		

Table S6. The list of nucleoside standards used in studies, molecular formula and UV characteristics.

Nucleoside standards	Molecular Formula	Monoisotopic Mass	UV characteristic, λ_{max}	Extinction coefficient ε [M^{-1} cm^{-1}]
**mcm5S2U**	$C_{12}H_{16}N_2O_7S$	332.06782	278 nm	15383
**cm5S2U**	$C_{11}H_{14}N_2O_7S$	318.05217	274 nm	9312
**S2U**	$C_9H_{12}N_2O_5S$	260.04669	274 nm	13100
**mcm5H2U**	$C_{12}H_{16}N_2O_7$	300.09575	247 nm	14008
**cm5H2U**	$C_{11}H_{14}N_2O_7$	286.08010	249 nm	11358
**H2U**	$C_9H_{12}N_2O_5$	228.07462	246 nm	7200
**mcm5U**	$C_{12}H_{16}N_2O_8$	316.09067	268 nm	5525
**cm5U**	$C_{11}H_{14}N_2O_8$	302.07502	268 nm	6844
**U**	$C_9H_{12}N_2O_6$	244.06954	268 nm	10100

Table S7. LC-MS/MS analysis conditions (A) LC gradient, (B) TOF MS scanning parameters, (C) TOF MS/MS scanning parameters .

A. LC gradient

Time [min]	Flow [mL/min]	Buffer A [%]	Buffer B [%]
0	0.5	98	2
1	0.5	95	5

4	0.5	40	60
5	0.5	40	60
5.1	1	98	2
6.1	0.5	98	2

B. TOF MS scanning parameters

	**POS**
TOF start mass *[m/z]*:	100
TOF stop mass *[m/z]*:	840
Spray voltage [V]:	5000
DP:	50
CE:	10
Accumulation time [s]	0.1
Time bins to sum	4

C. TOF MS/MS scanning parameters

**Analyte**	**Precursor ion *[m/z]***	**Fragment ion *[m/z]***	**TOF start [*m/z*]**	**TOF stop [*m/z*]**	**Acumulation time (s)**	**DP**	**CE**	**RT (min)**	**Sum time intervals**
S2U	261.0540	129.0115	50	270	0.05	35	17	2.25	4
H2U	229.0819	97.0394	50	240	0.05	35	17	1.32	4
U	245.0768	113.0345	50	255	0.05	31	17	1.41	4
mcmS2U	333.0751	201.0332	50	331	0.05	36	15	3.23	4
mcmH2U	301.1030	169.0611	50	310	0.05	26	17	2.00	4
mcm5U	317.0979	185.0556	50	330	0.05	40	17	2.61	4
cm5S2U	319.0595	187.0175	50	330	0.05	51	15	2.82	4
cm5H2U	287.0874	155.0453	50	295	0.05	46	17	1.41	4
cm5U	303.0823	171.0403	50	310	0.05	51	15	1.71	4

Figure S1. T7 Endonuclease assay for selected clones of modified HEK293 with damaged *SOD2* or *Cat* genes selected for genomic DNA sequencing.

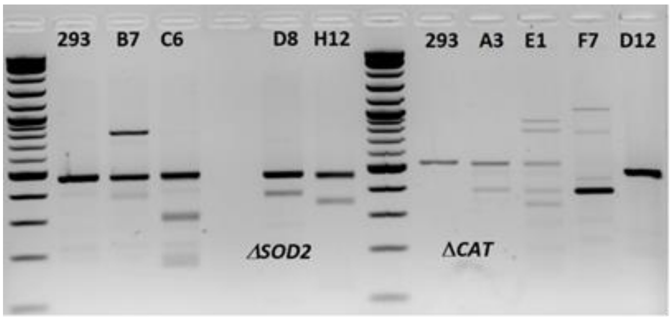

Figure S2. The result of sequencing of genomic DNA isolated from HEK293 cells with the damaged *SOD2* or *Cat* genes. Comparison of the sequences obtained from the modified clones (upper strand) with the original gene sequence (lower strand). The target sequences for the sgRNAs are marked in green. Sequence changes resulting from the CRISPR/Cas9 gene editing are marked in yellow.

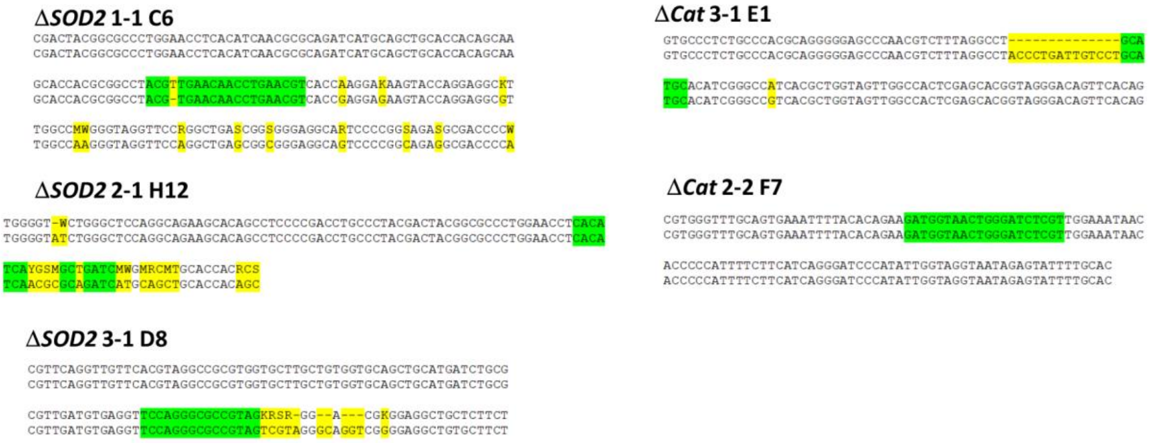

Figure S3. The results of the spotting test to investigate the viability of yeast cells exposed to oxidizing agents for 1 or 2 hours. The red line shows the maximum concentration of oxidizing agent that is safe for the cells. (A) Exposure to $NaAsO_2$ (0, 10, 20, 40, 80, 100 mM), (B) Exposure to H_2O_2 (0, 5, 10, 25, 50, 75, 100 mM), (C) Exposure to NaClO (0, 3, 4, 5, 7, 10 mM).

A. $NaAsO_2$ (0-100 mM)

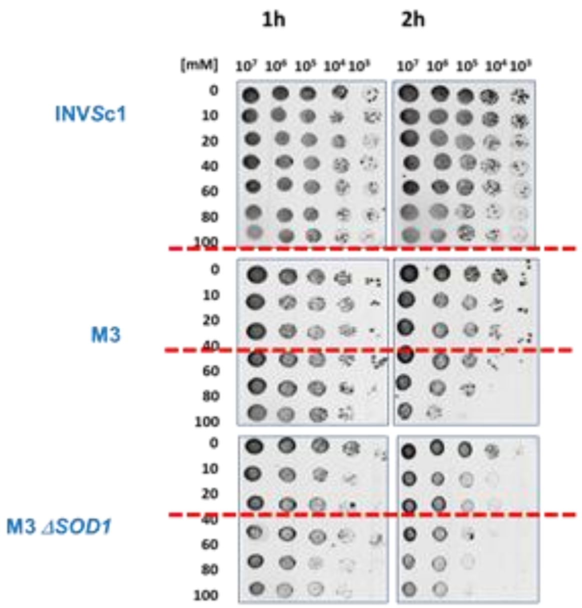

B. H_2O_2 (0-100 mM)

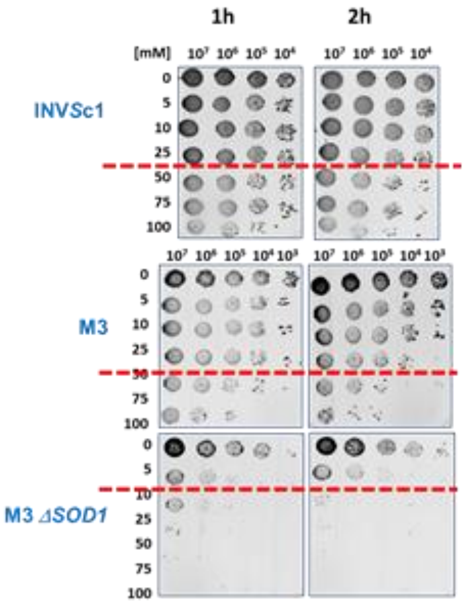

C. NaClO (0-10 mM)

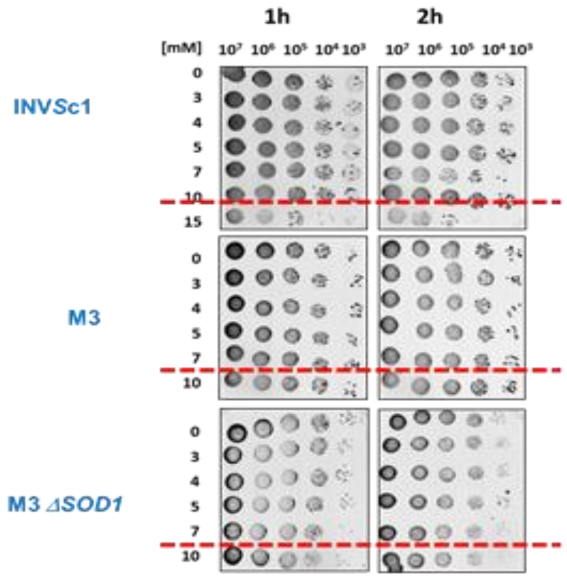

Figure S4. Determination of the ROS level in yeast cells after incubation with NaClO. SOD1 (-) indicates the M3*Δsod1* strain. The red rectangle indicates the ROS level formed under the influence of oxidizing agents at previously determined "safe concentrations" for yeast cells (red rectangle).

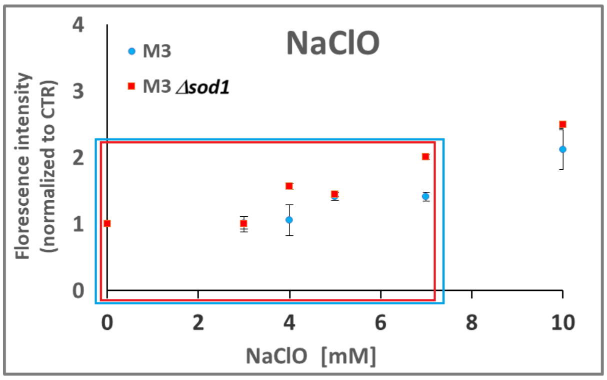

Figure S5. Visualization of ɣ-toxin (~27 kDa) protein in SDS-PAGE, gel stained with Coomassie Brilliant Blue dye. Line 1. protein mass standard, Line 2. purified protein.

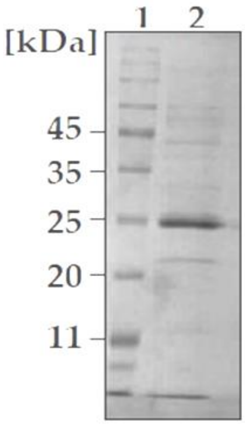

Figure S6. The MTT assay for cancer cells exposed to the oxidative reagents $NaAsO_2$ or NaClO. Evaluation of cell viability as a function of reagent concentration.

.

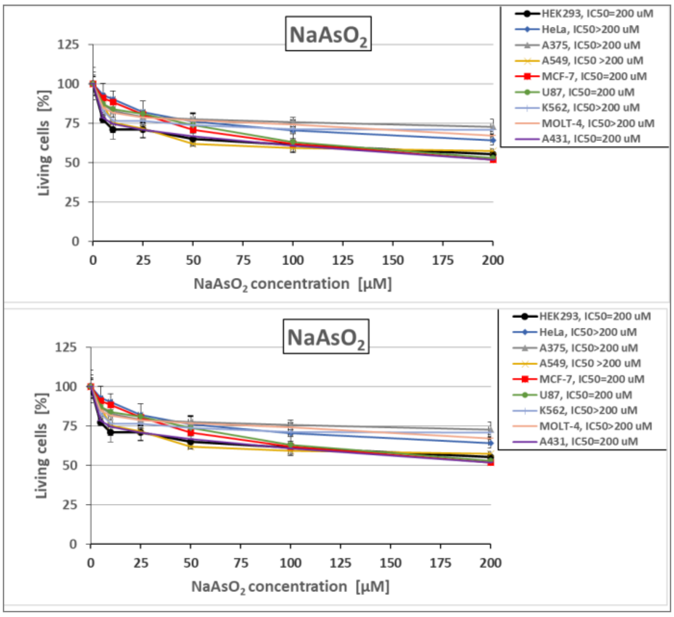

Figure S7. ROS generation in the cancer cells exposed to H_2O_2, $NaAsO_2$ or NaClO determined by 2,7 dichloro-dihydro-fluorescein diacetate assay.

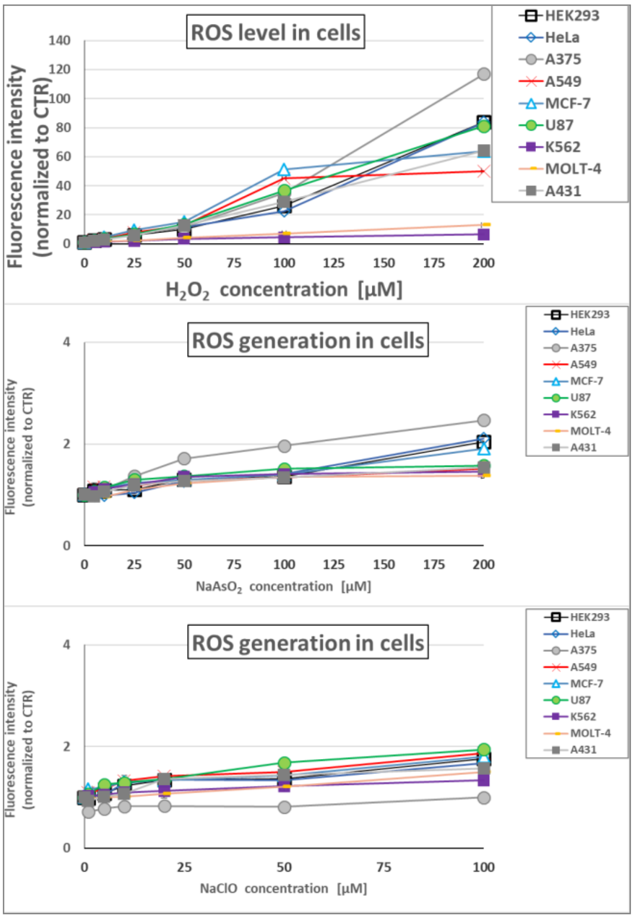

Figure S8. The natural intracellular ROS level existing in cancer cells, evaluated using fluorescein 2,7-dichloro-dihydro-diacetate assay.

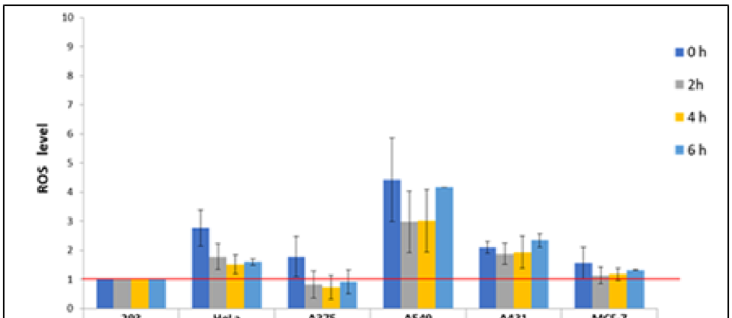

	293	HeLa	A375	A549	A431	MCF-7
0 h	1,00	2,76	1,78	4,42	2,09	1,56
2h	1,00	1,78	0,83	2,98	1,88	1,13
4 h	1,00	1,52	0,73	3,01	1,93	1,18
6 h	1,00	1,59	0,91	4,17	2,34	1,32

Figure S9. Structural formula of the nucleoside standards used in the LC-MS/MS (MRM) studies.

mcm5S2U	cm5S2U	S2U
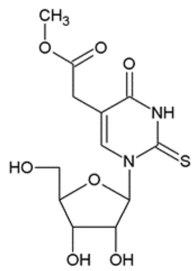	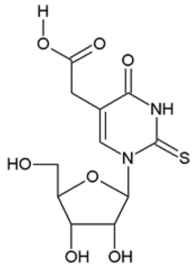	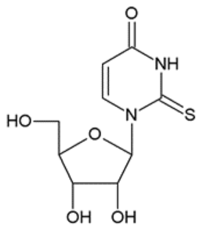
**mcm5H2U**	**cm5H2U**	**H2U**
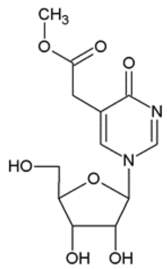	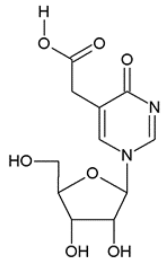	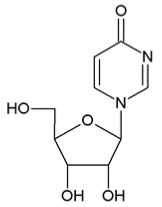
**mcm5U**	**cm5U**	**U**
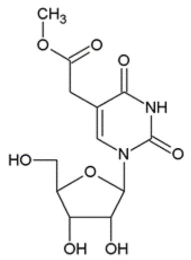	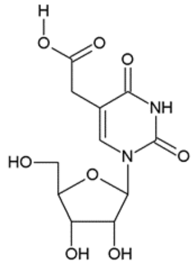	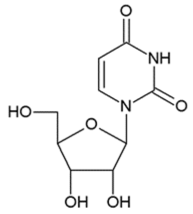

Figure S10. The extracted ions chromatogram (XIC) of the mixture of ten nucleoside standards (A, G, U, C, mcm5S2U, mcm5H2U, mcm5U, cm5S2U, cm5H2U and cm5U).

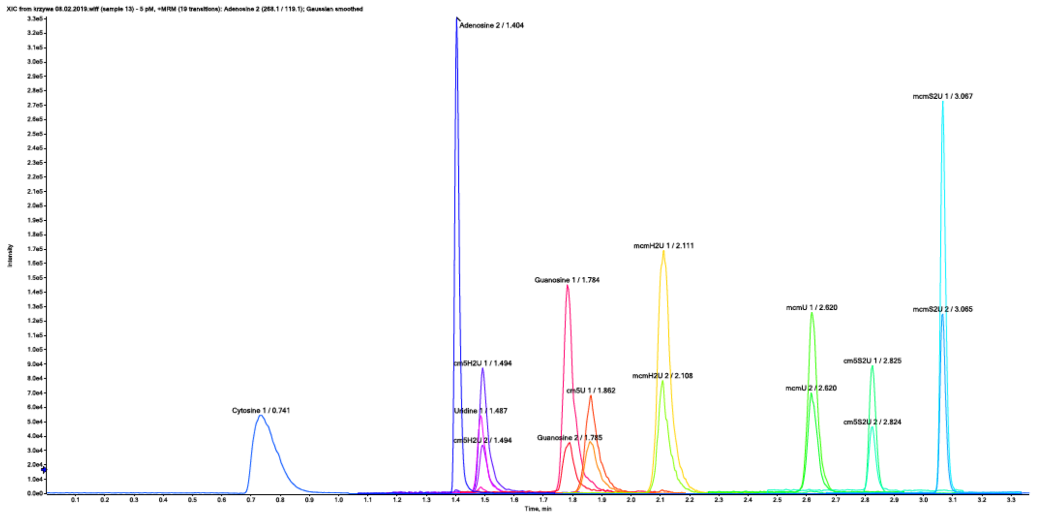

Figure S11. Standard curves prepared for nucleoside standards.

Created with SCIEX OS 3.0.0
Printed:11/14/2022 2:39:39 PM

Analyte Name: mcmS2U
Internal Standard: N/A

**Data File**	20221107_krzywa.wiff2	**Result Table**	20221108 krzywa
**Acquisition Date**	11/7/2022 3:47:38 PM	**Algorithm Used**	AutoPeak
**Acquisition Method**	N/A	**Instrument Name**	ZenoTOF™ 7600 System
**Project**	CBMM PAN	**Processing Method**	nukleozydy v1.qmethod

Regression Equation: y = 8510.35899 x + 17.35438 (r = 0.99965, r² = 0.99929) (weighting: 1 / x)

Expected Concentration	Number of Values	Mean Calculated Concentration (nM)	Average Accuracy across Replicates	Std. Deviation	%CV
0.01	3 of 3	0.011	106.7	0.00	13.5
0.05	3 of 3	0.050	100.8	0.01	17.3
0.10	3 of 3	0.090	90.3	0.01	6.3
0.50	3 of 3	0.480	96.0	0.01	2.3
1.00	3 of 3	1.008	100.8	0.03	2.8
5.00	3 of 3	5.017	100.3	0.20	3.9
10.00	3 of 3	10.108	101.1	0.25	2.5
50.00	3 of 3	51.281	102.6	0.80	1.6
100.00	3 of 3	100.627	100.6	1.44	1.4
500.00	2 of 3	513.656	102.7	3.51	0.7
1000.00	3 of 3	988.883	98.9	30.26	3.1

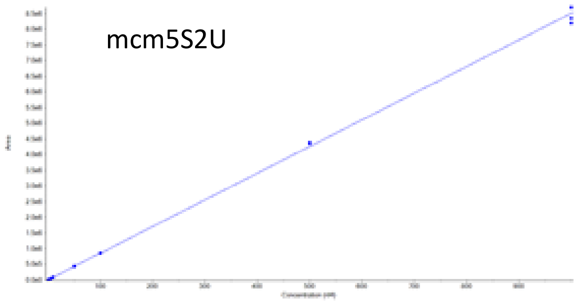

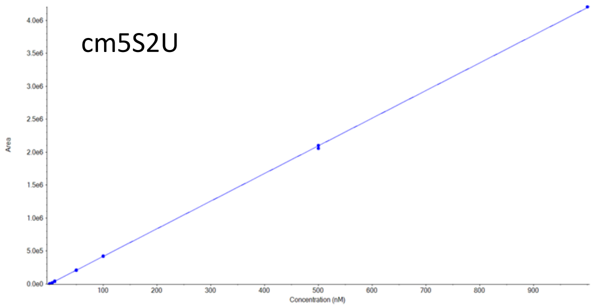

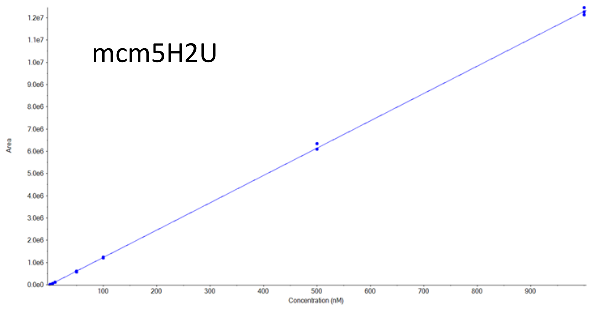

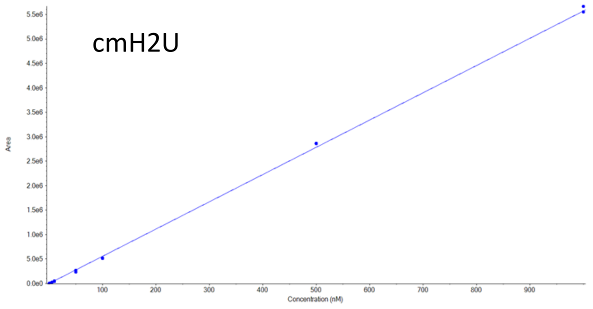

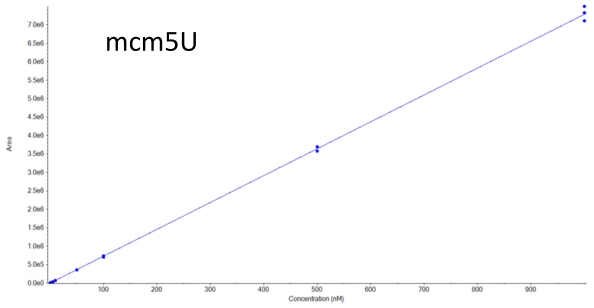

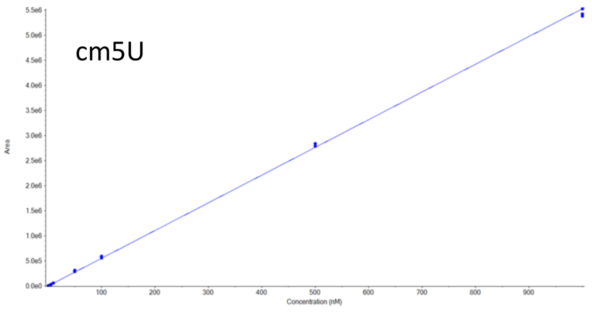

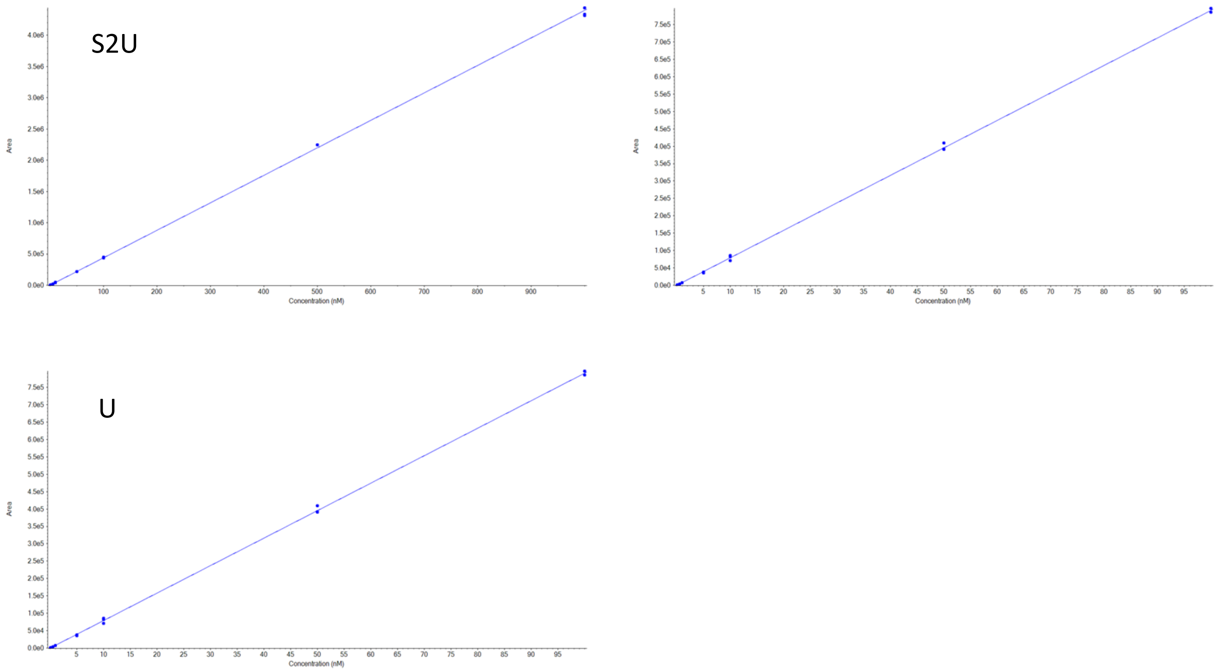

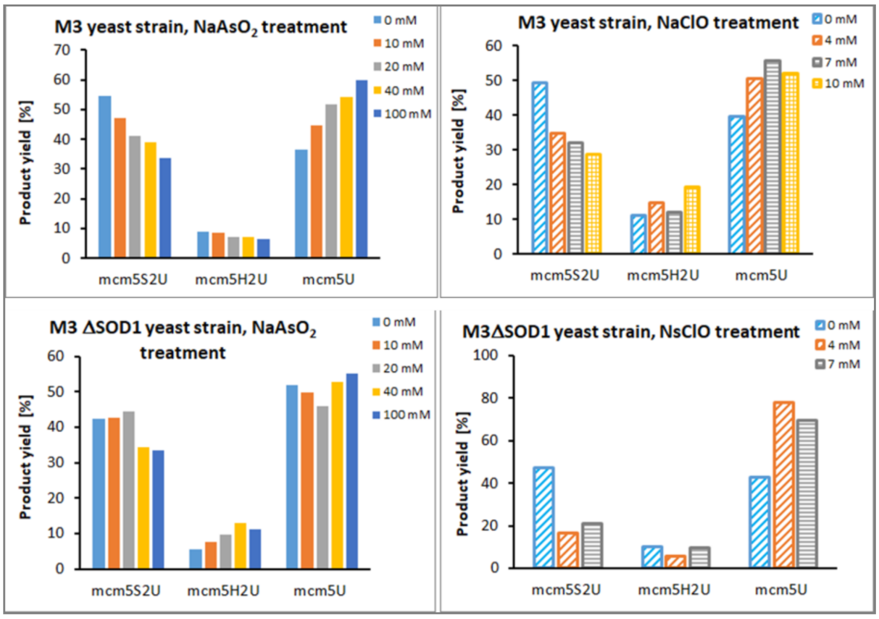

Figure S12. Identification of mcm5S2U desulfuration products in yeast tRNA^{Glu} by LC-MS/MS, yeast exposed to $NaAsO_2$ or NaClO during culture.
